# Mutational profiles of marker genes of cervical carcinoma in Bangladeshi patients

**DOI:** 10.1186/s12885-021-07906-5

**Published:** 2021-03-18

**Authors:** Shahana Sharmin, Fatima Tuj Zohura, Md. Sajedul Islam, Anika Shimonty, Md. Abdullah-Al-Kamran Khan, Rehana Parveen, Foujia Sharmin, Chowdhury Rafiqul Ahsan, Abul Bashar Mir Md. Khademul Islam, Mahmuda Yasmin

**Affiliations:** 1 Department of Microbiology, University of Dhaka, Dhaka, Bangladesh; 2 Department of Genetic Engineering and Biotechnology, University of Dhaka, Dhaka, Bangladesh; 3 Current Affiliation: Internal Medicine OPD, Bangabandhu Sheikh Mujib Medical University Hospital, Dhaka, Bangladesh; 4 Current Affiliation: Department of Biochemistry and Biotechnology, University of Barisal, Barisal, Bangladesh; 5 Current Affiliation Department of Mathematics and Natural Sciences, BRAC University, Dhaka, Bangladesh; 6 Square Hospital, Dhaka, Bangladesh; 7 Department of Gynecological Oncology, National Institute of Cancer Research & Hospital, Dhaka, Bangladesh

**Keywords:** Mutation, Cervical cancer, *EGFR*, *PIK3CA*, *KRAS*, HPV

## Abstract

**Background:**

Cervical cancer is a gynecologic cancer type that develops in the cervix, accounting for 8% mortality of all female cancer patients. Infection with specific human papillomavirus (HPV) types is considered the most severe risk factor for cervical cancer. In the context of our socioeconomic conditions, an increasing burden of this disease and high mortality rate prevail in Bangladesh. Although several researches related to the epidemiology, HPV vaccination, and treatment modalities were conducted, researches on the mutation profiles of marker genes in cervical cancer in Bangladesh remain unexplored.

**Methods:**

In this study, five different genomic regions within the top three most frequently mutated genes (*EGFR, KRAS* and *PIK3CA)* in COSMIC database with a key role in the development of cervical cancers were selected to study the mutation frequency in Bangladeshi patients. *In silico* analysis was done in two steps: nucleotide sequence analysis and its corresponding amino acid analysis.

**Results:**

DNA from 46 cervical cancer tissue samples were extracted and amplified by PCR, using 1 set of primers designed for *EGFR* and 2 sets of primers designed for two different regions of both *PIK3CA* and *KRAS* gene. In total, 39 mutations were found in 26 patient samples. Eleven different mutations (23.91%), twenty-four different mutations (52.17%) and four mutations (8.7%) were found in amplified *EGFR, PIK3CA* and *KRAS* gene fragments, respectively; among which 1 (*EGFR*) was common in seven patient samples and 2 (*PIKCA*) were found in more than 1 patient. Our study shows that except for *KRAS*, the frequency of observed mutations in our patients is higher than those reported earlier in other parts of the world. Most of the exonic mutations were found only in the *PIK3CA* and *EGFR* genes.

**Conclusions:**

The study can be used as a basis to build a mutation database for cervical cancer in Bangladesh with the possibility of targetable oncogenic mutations. Further explorations are needed to establish future diagnostics, personalized medicine decisions, and other pharmaceutical applications for specific cancer subtypes.

**Supplementary Information:**

The online version contains supplementary material available at 10.1186/s12885-021-07906-5.

## Background

Cervical cancer is a type of gynecologic cancer that develops in the cervix, which is a part of the uterus. There was a report estimating 528,000 new cases of cervical cancer, of which around 85% occurs in less developed regions. Moreover, around 266,000 deaths occur annually due to cervical cancer, accounting for 8% of all female cancer deaths [1]. In 2014, an estimated 12,578 women in the United States were diagnosed with cervical cancer; among them, 4115 women died [2]. The widespread use of cervical cancer screening programs has dramatically reduced the rates of cervical cancer in developed countries [3].

Infection with HPV is the most important risk factor for cervical cancer [4]. In humans, specific papillomavirus types have been associated with over 99% of cervical cancer biopsies [5]. It is a common virus that can be sexually transmitted. Sexually active women commonly harbor HPV infection which is highly contagious to their partner [6]. HPV belongs to papillomavirus family and is a small, icosahedral, non-enveloped DNA virus [7]. HPV show tropism for stratified squamous epithelium. More than 200 genotypes of HPV have been found and were classified as oncogenic and the non-oncogenic types [8]. Among the oncogenic viruses, only 15 are identified as high-risk types. They can cause neoplastic changes to the cervical epithelium. Globally, 75% of cervical cancer cases are caused by HPV types 16 and 18, while 31 and 45 are the causes of another 10% [9].

Besides HPV, multiple factors such as cigarette smoking (both active and passive), long-term use of oral contraceptives, multiple pregnancies, low socioeconomic status, being immunocompromised, multiple sexual partners are associated with increased risk of cervical cancer [10].

Cervical cancer, like other cancers, is a disorder of cell growth regulation. Mutation is a common change, which means any permanent changes in genomic DNA. Mutation may be missense, nonsense, insertion, deletion, duplication, frameshift mutation and repeat expansion [11]. These mutations are considered as significant when they occur in the genes which control different signal transduction pathways. There are specific genes which regulate cell growth and differentiation. Altered function of these genes leads a normal cell to transform into a cancer cell [12].

Among the high number of genes involved in different signal transduction and cell growth regulation pathways, some are of special interest. Mutations in these genes play a key role in the development of different cancers. These are *EGFR* (Epidermal Growth Factor Receptor), *KRAS* (Kirsten rat sarcoma), and *PIK3CA* (phosphatidylinositol-4, 5-bisphosphate 3-kinase, catalytic subunit alpha). The *EGFR* gene product of is a receptor for members of the epidermal growth factor family (EGF family) of extracellular protein ligands [13]. *KRAS* gene which is a proto-oncogene corresponding to the oncogene that was first identified in Kirsten rat sarcoma virus [14] and its protein product is a GTPase that is an early player in many signal transduction pathways. Protein product of *PIK3CA* (phosphatidylinositol-4, 5-bisphosphate 3-kinase, catalytic subunit alpha) gene uses ATP to phosphorylate phosphatidylinositols (PtdIns), PtdIns4P and PtdIns P2.

In the context of low socioeconomic condition, we are experiencing an increasing burden of cervical cancer and mortality rate is quite high. To best of our knowledge, any extensive research on the mutation profiling of cervical cancer affected patients in Bangladesh has not done yet. Without this information, decision of chemotherapy is most cases difficult and become non-specific treatment. The aim of this study was to find out mutation of any of these genes in cancerous tissue of cervical carcinoma patients in Bangladesh and to rule out the significance of these mutations in developing the disease as well.

## Methods

### Sample collection

Cervical tissue samples were collected from cervical cancer patients from the National Institute of Cancer Research and Hospital (NICHR), Mohakhali, Dhaka and Bangabandhu Sheikh Mujib Medical University (BSMMU), Shahbag, Dhaka between February 2015 and June 2018 if they satisfied the following conditions: pathologically determined primary cervical carcinomas, stages IA–IIB according to the 2014 International Federation of Gynecology and Obstetrics (FIGO) staging system, and no prior neoadjuvant chemotherapy or radiation. The specimens were collected during radical hysterectomy procedures and specimens were kept at -20 °C in RNAlater solution (Ambion; Thermo Fisher Scientific, Waltham, MA, USA) until processing. All the specimens were squamous cell carcinoma. Both institutional ethical clearance (IRB, Bangladesh) and patients’ written consents were taken prior to sample collection.

### Tissue sample processing

Genomic DNA was extracted from the cervical tissue samples using QIAamp® DNA Mini Kit (QIAGEN, Germany). Quality and quantity of the extracted DNA were analyzed using gel electrophoresis and NanoDrop™ spectrophotometer respectively.

### Detection of mutations in the target genes

Findings of Wright et al. reflect previous studies that shows high mutation rates in *PIK3CA* in cervical cancer [15]. *KRAS* and *EGFR* mutations in cervical cancer were also reported in many studies [15]. In COSMIC database [16], *PIK3CA*, *KRAS* and *EGFR* are ranked top 3 among 20 genes that have high mutation rates in cervical cancer. So, these 3 genes were selected as the targets for our study, and mutation hotspots were amplified using specific PCR primers.

*EGFR* gene fragments were amplified using one set of primers for each gene. Since *PIK3CA* and *KRAS* genes were indicated to harbor more mutation hotspot than *EGFR*, two sets of primers were used to amplify two different regions of the genes (Table 1). In total, forty-six extracted DNA samples were used in this study.

**Table 1:** Primers used for detection of gene mutation

Target Gene	Primer Sequence		Amplicon Size (bp)	GC%
***PIK3CA*_1**	F	AGACAATGAATTAAGGGAAAATGAC	690	32%
	R	GTTATACCACTCTTCATATAGCTCA		36%
***PIK3CA*_2**	F	AGTGGGGTAAAGGGAATCAAAAGA	533	41.7%
	R	GCAATTCCTATGCAATCGGTCT		45.5%
***EGFR***	F	ACGAAGCCTGTGTGTTTGGT	588	50%
	R	GGCAAAGGGAGTGGAAGGAA		55%
***KRAS*_1**	F	CTTAAGCGTCGATGGAGGAGT	545	52.4%
	R	ACCCTGACATACTCCCAAGGA		52.%
***KRAS*_2**	F	TGTCCGTCATCTTTGGAGCA	549	50%
	R	TTTCAATCCCAGCACCACCA		50%

(R = Reverse primer, F=Forward primer. Suffix with an underscore after the gene name indicates primer set number)

The PCR products were resolved in 1.5% agarose gel to analyze the specific band of the amplicon. Amplified fragments were excised from the agarose gel and purified using PureLink PCR Purification Kit (Invitrogen, USA). The purified DNA fragments were sequenced by a cycle sequencing strategy, using BigDye Terminator v3.1 Cycle Sequencing Kit (Applied Biosystems, USA).

### Detection and genotyping of associated HPV infections

For detection of HPV, conserved L1 region (encodes for major capsid protein) was targeted for PCR. In this study, firstly DNA fragment in the L1 region was amplified by using primer pair MY11/MY09 which would give a product of ~ 450 bp (Table 2). Then the product of MY-PCR was amplified by nested PCR using GP5/GP6 primer set which would give a product of ~ 150 bp (Table 2). To identify the genotype of the associated HPV, we performed PCR using the genotype-specific primer pairs for HPV-16 and HPV-18 genotypes (Table 2). Then we sequenced the PCR products following the direct sequencing strategy mentioned in the previous section. Finally, we did BLASTn [20] searches with the sequences which were amplified using the genotype specific primers to obtain the associated HPV genotype.

**Table 2:** Primers used for detection of HPV targeting L1 region

Primers	Primer Sequence	Amplicon size (bp)	Annealing temperature (°C)	References
MY11 (forward)	GCMCAGGGWCATAAYAATGGG	450	55	Sin Hang Lee, 2012 [17]
MY09 (reverse)	CGTCCMARRGGAWACTGATC			
GP5 (forward)	TTTGTTACTGTGGTAGATAC	150	49.2	de Roda Husman et al., 1995 [18]
GP6 (reverse)	GAAAAATAAACTGTAAATCA			
HPV-16 specific forward (inner)	TACCTACGACATGGGGAGGA	194	55	Ge et al., 2012 [19]
HPV-16 specific reverse (inner)	GCAATTGCCTGGGATGTTAC			
HPV-18 specific forward (inner)	TGGTGTTTGCTGGCATAATC	339	55	Ge et al., 2012 [19]
HPV-18 specific reverse (inner)	GCAGCATCCTTTTGACAGGT			

Key to degenerate nucleotides: M = (A + C), R = (A + G), W = (A + T), Y = (C + T)

### *In silico* analysis in search of potential mutations and their effects in the target genes

After gene fragment sequences were obtained, these were carefully analyzed in order to detect possible mutation. For this reason, sequence of each strand was separately compared to the NCBI GenBank [21] database using the BLASTn [20]. Additionally, types of the mutations found were categorized as well. Exonic mutations were determined and the frequencies of every exonic mutations were also calculated to find out the gene which contains the most exonic mutations.

Ensembl Variant Effect Predictor (VEP) [22] tool was applied in search of the effects of the variants of the mutations across Ensembl and RefSeq transcripts databases. LoFtool [23] was used in order to find out the intolerance score based on loss-of-function of variants of the mutation. In order to find out the effects of missense mutations on the protein level, two different scores, SIFT [24] and Polyphen [25] scores, were estimated from the integrated calculation of the VEP tool. MuPro tool [26] was also used which uses sequence and structure related information in order to distinguish the effects of mutations on protein level.

### Statistical analysis

We performed Fisher’s exact test to find the association between the different gene mutations and the several clinicopathological features of the patients. These analyses were carried out using the IBM SPSS Statistics software v20 (IBM Corporation, New York, NY, USA). The results were considered statistically significant when the associated *p*-value was smaller than 0.050.

## Results

### Molecular detection of specific gene fragments in cervical cancer samples by polymerase chain reaction (PCR)

**Fig. 1:**
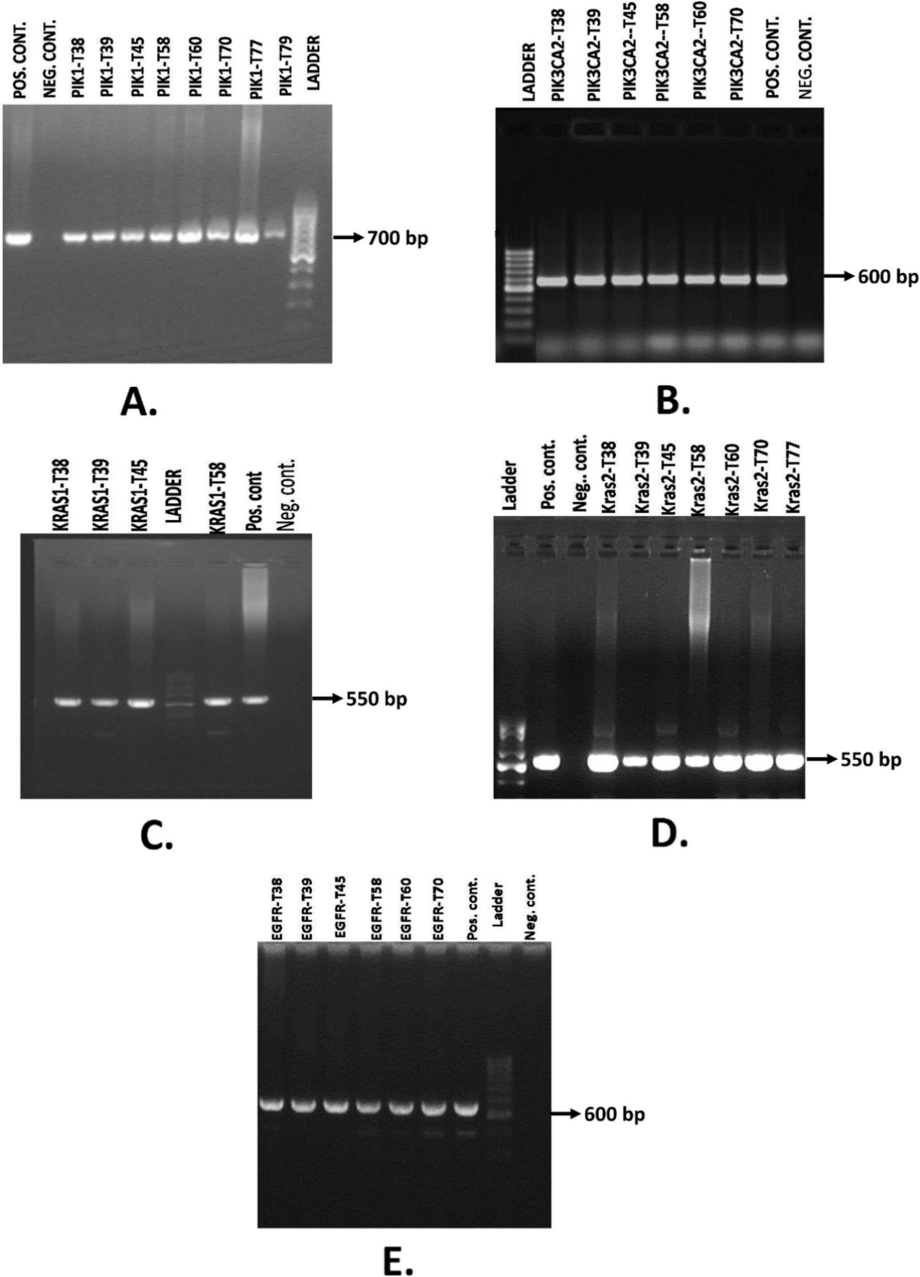
Agarose gel electrophoresis of PCR specific amplicon using **a.** PIK3CA_1, **b.** PIK3CA_2, **c.** KRAS_1, **d.** KRAS_2, **e.** EGFR primer pairs. 50 bp ladder (Bioneer, USA) was used for comparison

Among all the samples that we have got after the histopathology test it has revealed that 2 adenocarcinoma cases and all other cases are squamous cell carcinomas. Afterward PCR was carried out for amplifying target gene fragment for 46 patients’ samples (Supplementary file 1). Using the PIK3CA_1 and PIK3CA_2 primer sets, PCR products were estimated to be sized approximately 700 bp and 600 bp, respectively on agarose gel electrophoresis (Fig. 1a-b). While using the KRAS_1 and KRAS_2 primer sets, PCR products showed band on agarose gel electrophoresis at approximately 550 bp for both (Fig. 1c-d). PCR products obtained using *EGFR* primer sets were sized at approximately 600 bp on agarose gel electrophoresis (Fig. 1e).

In a total, 230 gene fragments were sequenced (both strands). Using BLAST search, 39 mutations were found in 26 patients among the 46 patient samples. The corresponding chromatograms of sequenced fragments were carefully inspected to ensure that the base change is not due to a sequence slippage but a valid mutation. If a clear peak was detected denoting one specific base, the sequence was acknowledged to be correct and base change was counted. Base changes for which clear single peaks did not exist were not counted as mutation. Mutation was confirmed from the chromatogram of second strand that was also sequenced. The rest of the samples did not harbor any mutation along the region we sequenced.

**Fig. 2:**
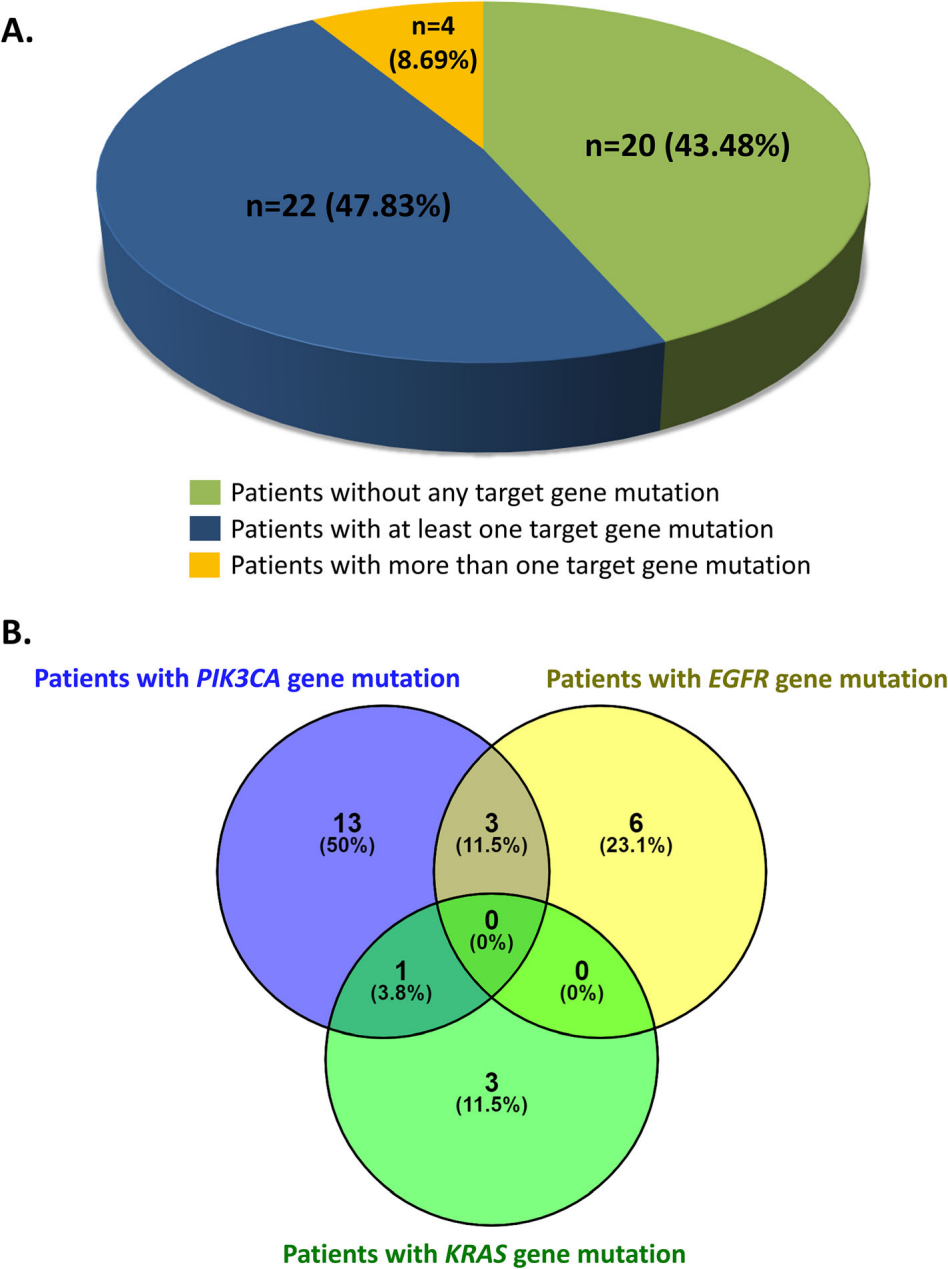
**a.** Distribution of cases based on the presence of number of mutation(s) within the target genes. **b.** Venn diagram indicating the percentages of the patients with single gene mutation and multiple gene mutations

In summary, our data show that among 46 samples, 20 samples did not harbor any mutation (43.48%), 22 samples harbored mutation in at least one gene fragment (47.83%) whereas 4 samples harbored mutations in more than one gene fragments (8.69%) (Fig. 2 a-b).

We observed that most the patients were aged in between 30 and 45 years (*n* = 26, 56.52%), while the other patients were more than 45 years old (*n* = 20, 43.48%) (Table 3). The median age of this patient cohort was 45 years with the maximum of 78 years and minimum of 35 years. We also recorded that most of the patients were infected with HPV-16 (*n* = 21, 45.65%), while the others were found infected with both HPV-16 and 18 (*n* = 17, 36.96%) and only HPV-18 (*n* = 7, 15.22%) (Table 3). One of the patients did not have any HPV infections (2.17%) (Table 3). Disease stage of the most patients were either FIGO stage IA (*n* = 14, 30.43%) or stage IB (*n* = 20, 43.48%) (Table 3). Rest of the patients’ cancer stage progressed further into the FIGO stage IIA (*n* = 8, 17.39%) and stage IIB (*n* = 4, 8.7%) (Table 3). However, we did not observe any significant association between the individual/overall gene mutations of the patients and some clinical features of the patients (Table 3).

**Table 3:** Association between the target gene mutations and several clinicopathological variables

**For** ***KRAS*** **gene mutations only**				
**Variables**	**Cases**	**Wild Type (*n*** **= 42)**	**Mutant (*n*** **= 4)**	***p*-value (Fisher’s exact test)**
**Age (yr)**				
30–45	26	25	1	0.303
> 45	20	17	3	
**HPV infections**				
HPV 16	21	18	3	0.672
HPV 18	7	7	0	
HPV 16 & 18	17	16	1	
Negative	1	1	0	
**FIGO stage**				
IA	14	11	3	0.136
IB	20	20	0	
IIA	8	7	1	
IIB	4	4	0	
**For** ***PIK3CA*** **gene mutations only**				
**Variables**	**Cases**	**Wild Type (*n*** **= 29)**	**Mutant (n = 17)**	**p-value (Fisher’s exact test)**
**Age (yr)**				
30–45	26	15	11	0.538
> 45	20	14	6	
**HPV infections**				
HPV 16	21	15	6	0.480
HPV 18	7	3	4	
HPV 16 & 18	17	10	7	
Negative	1	1	0	
**FIGO stage**				
IA	14	8	6	0.788
IB	20	13	7	
IIA	8	6	2	
IIB	4	2	2	
**For** ***EGFR*** **gene mutations only**				
**Variables**	**Cases**	**Wild Type (*n*** **= 37)**	**Mutant (*n*** **= 9)**	**p-value (Fisher’s exact test)**
**Age (yr)**				
30–45	26	20	6	0.711
> 45	20	17	3	
**HPV infections**				
HPV 16	21	17	4	0.391
HPV 18	7	6	1	
HPV 16 & 18	17	14	3	
Negative	1	0	1	
**FIGO stage**				
IA	14	11	3	0.291
IB	20	14	6	
IIA	8	8	0	
IIB	4	4	0	
**Considering all gene mutations**				
**Variables**	**Cases**	**Wild-Type (n = 20)**	**Mutant (n = 26)**	**p-value (Fisher’s exact test)**
**Age (yr)**				
30–45	26	11	15	> 0.999
> 45	20	9	11	
**HPV infections**				
HPV 16	21	10	11	0.955
HPV 18	7	3	4	
HPV 16 & 18	17	7	10	
Negative	1	0	1	
**FIGO stage**				
IA	14	4	10	0.480
IB	20	9	11	
IIA	8	5	3	
IIB	4	2	2	

Amongst the 46 samples, it has been found that *PIK3CA* was the most frequently mutated gene (in 52.17% patients), *EGFR* gene was the second most mutated gene (in 23.91% patients) and finally *KRAS* gene was found to be the least mutated in the tested patients (in 8.7% patients) (Table 4).

**Table 4:** Percentage of mutation found in the target genes

***KRAS*** Gene	***EGFR*** Gene	***PIK3CA*** Gene
4 in 46 samples (**8.7%)**	11 in 46 samples (**23.91%)**	24 in 46 samples (**52.17%)**

### Mutations in *PIK3CA* and *EGFR* genes were found to be more prevalent in Bangladeshi patients compared to previously conducted study

**Fig. 3:**
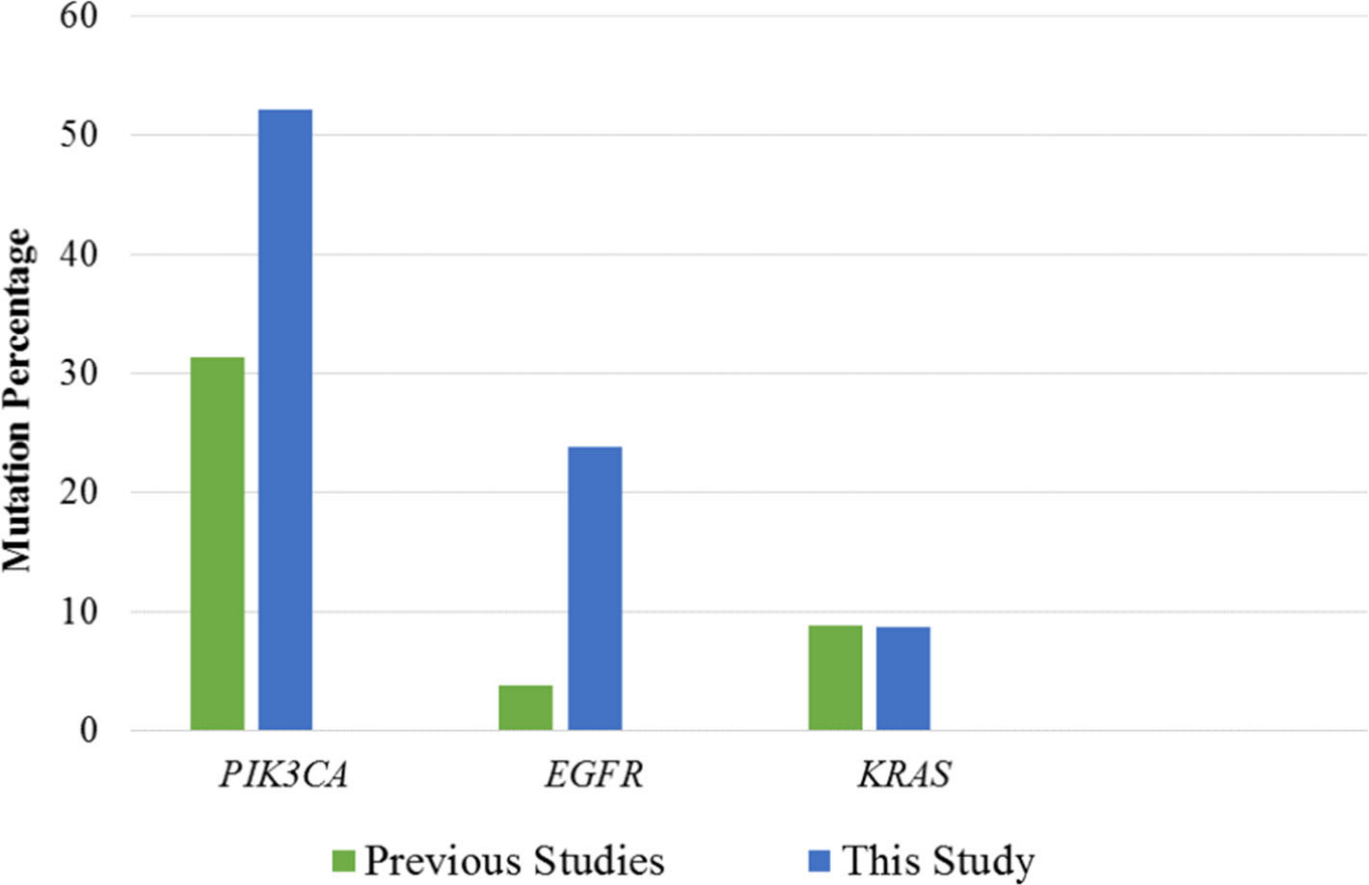
Comparison of gene specific mutation percentages between previous work of Wright et al., 2013 and our present study

Some studies have been done on individual gene mutations in cervical cancer in other parts of the world. Study of Wright et al. [15], have been compared with this study (Fig. 3). A much higher percentage of *PIK3CA* mutation was observed in this study compared to the previous one (52.17% in this study vs. 31.3% in the previous study). Similarly, *EGFR* mutation rate was found to be much prevalent in Bangladeshi patients compared to the statistics of Wright et al. [15] (23.91% in this study vs. 3.8% in the previous study). However, not much of a difference has been observed for the *KRAS* gene mutation (8.7% in this study vs. 8.8% in the previous study) (Fig. 3).

### Moderate amount of exonic mutations were found only in *PIK3CA* and *EGFR* genes

Among the observed mutations, 17 different mutations were found to be unique as each of them occurs in a specific chromosomal location. These 17 mutations were analyzed using Variant Effect Predictor tool [22]. All these different mutations in the 3 target genes showed significant low LoFtool [23] scores (Table 5) which can be used to predict that all these mutations can have probable damaging effects to make the patients more vulnerable to carcinogenesis and metastatic development.

**Table 5:** Putative effect of the mutation on gene function based on LoFtool predictions

Gene	Mutation	Intron/ Exon	Position (Chromosome)	LoFtool Score	Mutation Effect
*EGFR*	G > A	Exon	Chr7 55,170,575	0.0455	Probably Damaging
	G > A	Intron	Chr7 55,170,193	0.0455	Probably Damaging
	G > A	Intron	Chr7 55,170,210	0.0455	Probably Damaging
	Insertion (A)	Intron	Chr7 55,170,230	0.0455	Probably Damaging
	A > G	Exon	Chr7 55,170,332	0.0455	Probably Damaging
*KRAS*	G > A	Intron	Chr12 25,245,246	0.19	Probably Damaging
	A > C	Intron	Chr12 252,271,121	0.19	Probably Damaging
*PIK3CA*	T > A	Exon	Chr3 179,218,237	0.268	Probably Damaging
	T > A	Exon	Chr3 179,234,443	0.268	Probably Damaging
	C > T	Intron	Chr3 179,218,425	0.268	Probably Damaging
	T > G	Intron	Chr3 179,218,439	0.268	Probably Damaging
	G > A	Intron	Chr3 179,218,352	0.268	Probably Damaging
	T > A	Intron	Chr3 179,234,011	0.268	Probably Damaging
	C > T	Exon	Chr3 179,234,223	0.268	Probably Damaging
	T > A	Exon	Chr3 179,234,446	0.268	Probably Damaging
	T > A	Exon	Chr3 179,234,447	0.268	Probably Damaging
	C > T	Exon	Chr3 179,234,232	0.268	Probably Damaging

Eight mutations were found to be exonic among these 17 mutations and rest others were intronic. Among the exonic mutations, two mutations were found to be non-synonymous, one was found in the *PIK3CA* gene and other one in the *EGFR* gene. None of the mutations in the *KRAS* gene were exonic. *PIK3CA* gene was found to contain a high amount of exonic mutations (60%) than the *EGFR* gene (40%) (Table 6).

**Table 6:** Frequency of the exonic mutations found in the target genes

Gene	Total mutation	Exonic mutation	Number of non-synonymous mutation	Intronic mutation	% of exonic mutation
*PIK3CA*	10	6	1 out of 6	4	60%
*EGFR*	5	2	1 out of 2	3	40%
*KRAS*	2	0	0 out of 2	2	0%

### Resultant mutant amino acids of *PIK3CA* and *EGFR* proteins are found to be located in their functional domains

**Fig. 4:**
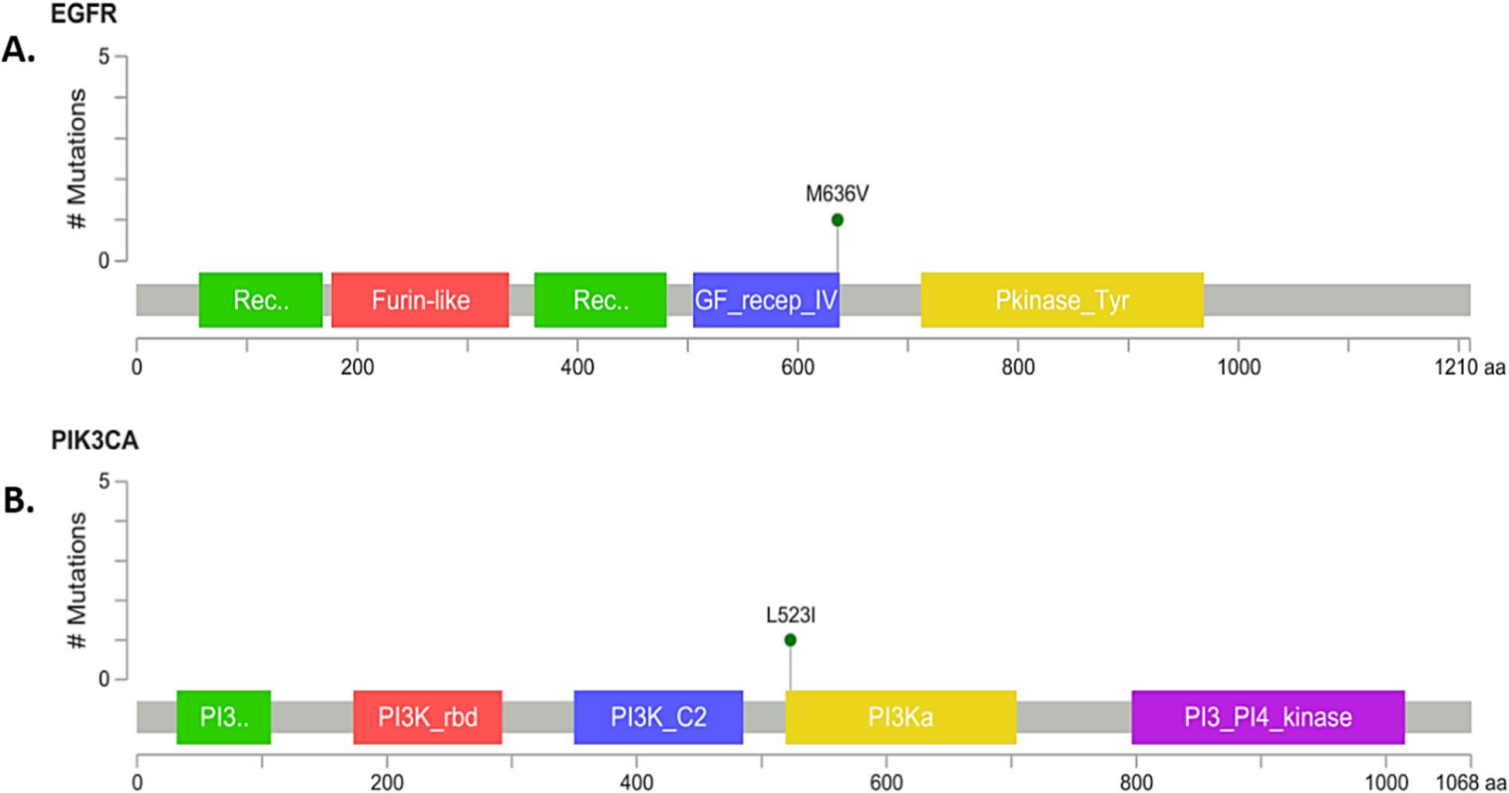
Location of the mutant amino acid in the **a.** EGFR protein structure, **b.** PIK3CA protein structure

Using MutationMapper tool [27] the particular domain where the target mutant amino acid was identified. In EGFR protein, mutant amino acid (valine) is present in a functional domain named Growth Factor Receptor 4 domain (Fig. 4a). Missense mutation of PIK3CA protein was located in the beginning of the PIK domain (Fig. 4b), which is conserved in all PI3 and PI4-kinases and role of this domain unclear but it has been suggested to be involved in substrate presentation [28].

### Non-synonymous mutations found in the *PIK3CA* and *EGFR* genes may lead to the production of associated nonfunctional proteins

SIFT [24] scores for the non-synonymous mutations are found to be high and PolyPhen [25] scores are found to be lower for both genes (Table 7), predicting the structure and function of the proteins can retain its natural states even though the mutation occur to the change the particular amino acid.

**Table 7:** Prediction of the effect of the non-synonymous mutation on protein structure and function

Gene	Mutation	Type	Position (Chromosome)	SIFT Score	PolyPhen Score
*EGFR*	G > A	Exonic	Chr7 55,170,575	0.5 (tolerated)	0 (benign)
*PIK3CA*	T > A	Exonic	Chr3 179,218,237	0.26 (tolerated low confidence)	0.001 (benign)

Due to the low delta G value for the both of the non-synonymous mutations, MuPro tool [26] predicted that both of the protein structures may lose its stability upon the accumulation of the particular variant amino acid instead of the wild-type one (Table 8).

**Table 8:** Prediction of protein’s stability upon the mutations

Gene	Mutation	Type	Position (Chromosome)	MuPro Score (∆G value)	MuPro Prediction
*EGFR*	G > A	Exonic	Chr7 55,170,575	−1.2135263	Decrease stability of protein structure
*PIK3CA*	T > A	Exonic	Chr3 179,218,237	−1.0116756	Decrease stability of protein structure

## Discussion

Cervical cancer is one of the most frequent cancer of women in 2012 representing 7.9% of all female cancers [29]. Approximately 90% of the 270,000 deaths from cervical cancer in 2015 occurred in low and middle-income countries [29]. This is the most common cause of female death by cancer in 43 countries. The estimated global economic burden of cervical cancer in 2009 was 3 billion US dollars.

Cancer causing infections, such as hepatitis and human papilloma virus (HPV), are responsible for up to 25% of cancer cases in low- and middle-income countries. Vaccination against these HPV and hepatitis B viruses could prevent 1 million cancer cases each year [30]. HPV is recognized as the most important risk factor for cervical cancer for last 15 years [31]. World health organization recognized sexually transmitted HPV infection as a modifiable and avoidable risk factor.

Bangladesh has a population of 54.38 million women ages fifteen years and older who are at risk of developing cervical cancer. Current estimates indicate that every year 11,956 women are diagnosed with cervical cancer and 6582 die from the disease [6]. Cervical cancer ranks as the second most frequent cancer among women between 15 and 44 years of age in Bangladesh [32]. In Southern Asia, the region Bangladesh belongs to, about 7.9% of women in the general population are estimated to harbor cervical HPV infection at a given time and 82.8% of invasive cervical cancers are attributed to HPV 16 or 18 [33].

Mutation that can induce neoplastic transformation is known as oncogenic mutation and can produce an abnormal protein or change expression level of the protein coded by the gene harboring the mutation. This abnormal or over expressed protein can be a potential target of drugs. This study attempted to identify mutations in genes which can be specific for cervical cancer.

In this study, we amplified specific gene fragments. *EGFR*, *KRAS* and *PIK3CA* genes were selected to be amplified in specific regions since these genes are implicated in most cancers, especially in cervical cancers. Total 5 pairs of primers were used. Two different sets of primers were chosen to amplify two different sites on *PIK3CA* and *KRAS* genes since these two genes were to harbor more than one mutation hotspot.

We observed that, among 46 samples, 26 samples harbored mutation (56.52%). The other samples did not harbor any mutation. Among the 26 samples which harbored mutation, 22 samples harbored mutation in one gene (84.62%) whereas 4 samples harbored mutations in more than one gene (15.38%).

Eleven different mutations were found in amplified *EGFR* gene fragments, among which 1 specific mutation was found in more than one patient samples (in 7 samples). On the other hand, 24 different mutations were found in *PIK3CA* gene fragment amplicons, among which 2 different mutations were found in 6 patients. Four different mutations were found in *KRAS* gene fragment amplified products.

Almost all the mutations identified were base substitution. Both transitions and transversions were found. Only one insertion mutation was found in *EGFR* gene fragment at chromosomal position 55,170,230 where an adenine residue was found added.

Our study shows that Bangladeshi patients have *KRAS* mutation frequency (8.7%) similar to that reported by Wright et al. [15] (8.8%). A 7% mutation in *KRAS* was also reported by Spaans et al., 2015 [34]. Iida et al., 2011 [35] also reported somatic mutations in *KRAS* in 3 (6.3%) of 48 cervical adeno/adenosquamous cell carcinomas.

However, surprisingly, *EGFR* mutation frequency is over 6 times higher in our patients (23.91% versus 3.8%) and *PIK3CA* mutation frequency is over 1.6 times (52.17% versus 31.3%) compared to study of Wright et al. [15], 2013 and 2.6 times higher (52.7% versus 20%) compared to Spaans et al., 2015 [34], report. Even *PIK3CA* mutation rate is 3.8 times higher than those in Chinese patients (13.6%) as reported by Xiang et al., 2015 [36].

Survival of patients with *KRAS* mutation is poorer than in patients without *KRAS* mutations [37], therefore, a combination of *KRAS* mutation detection and HPV genotyping would be useful in identifying a patient with poor prognosis for further interventions. Among the three most common histological subtypes of cervical cancer (squamous cell carcinoma (SCC), adenocarcinoma (AC), and adenosquamous carcinoma (ASC)) *KRAS* mutations are reported to occur more frequently in AC than SCC [34]. The same study also observed worse disease-free survival (HR 1.57, *P* = 0.043) in positive *KRAS* mutation cases. A set of endometrial-like cervical cancers comprised predominantly of HPV-negative tumors and characterized by mutations in *KRAS*, *ARID1A* and *PTEN* was discovered in another study [38].

EGFR is a membrane tyrosine kinase receptor that is known to contribute to the growth activity and tumor survival, and hence this has become a therapeutic target in several cancers. In *EGFR* gene, exons 18–21 are the hot spot region for gain-of-function mutations. Previous studies [35, 39] found a strong correlation between poor prognosis and *EGFR* gene amplification in patients with cervical squamous cell carcinoma. In other carcinomas like leukemia, glioblastoma, and colorectal, gastric, breast, and hepatocellular carcinomas *EGFR* mutation frequency reported to be low. Neither Iida et al., 2011 [35], nor the study of Arias-Pulido et al., 2008 [39] found presence of *EGFR* mutations in exons 19 and 21. No mutations identified in their samples affecting the EGFR kinase domain in exons 18 through 21 in human neoplastic samples analyzed. However, in cervical carcinoma, we have found a mutation in our Bangladeshi patients. These suggest that mutations in the EGFR kinase domain may be not common in other part of the world. Our results suggest, therefore, that treatment of CC patients with TKIs needs mutational screening before prescribing drugs and may not have the same efficacy as seen in patients with no-mutation. Therefore, CC patients without such mutation, targeting the *EGFR* with other inhibitors may be more appropriate.

PIK3CA mutations can cause the deregulation of the PI3K/Akt signaling pathway, which comprises cell proliferation, transformation, and cell survival, stimulating oncogenesis. Aberrations in this pathway are described in various cancers, including cervical cancer, and this has led to the development of PI3K-inhibitors and Akt-inhibitors as potential cancer therapies, with some already having reached clinical trials. *PIK3CA* mutation rates are very heterogeneous in different studies (20–37%) [40, 41]. However, Bangladeshi patients harbor more mutations (52.17%) in this gene. Spaans et al., 2015 [34] detected a clear trend for reduced survival in patients carrying a *PIK3CA* mutation, especially with the SCC subtype. Similarly Wright et al., 2013 [15] also showed that an association lies between *PIK3CA* mutation and shorter survival.

After DNA sequence analysis, corresponding amino acid analysis was done to find out the effect of mutations on proteins. Among the 17 different mutations, 8 mutations were located in exon regions. Six of them were *PIK3CA* mutations and the other 2 were *EGFR* gene mutations. The other 9 mutations were located in introns; hence, they did not have any effect on the overall amino acid sequence compositions.

These eight exonic mutations were analyzed and it was found out that 6 of them were synonymous. Among the rest 2 non-synonymous mutations, one mutation (A > G) in *EGFR* gene located at 55,170,332 in chromosome 7 results in a methionine to valine substitution at amino acid position 636 of the protein; while the result of the non-synonymous mutation (T > A) in *PIK3CA* gene located at 179,218,237 in Chromosome 3 is a substitution of leucine to isoleucine at amino acid position 523 of the protein.

We have done different bioinformatic analysis of mutations we have found using tools like Variant Effect Predictor tool [22], MuPRo tool [26], MutationMapper tool [27] etc. LoFtool [23] scores of all intronic and exonic mutations which were significantly low, predicts that effect of the mutation on the functionality of the gene is probably damaging. SIFT [24] and polyphen [25] scores showed that effect of the non-synonymous mutation on the function of protein is not that much significant. But the stability of protein may be affected which is predicted by MuPRo tool [26]. Further in vitro protein level analysis needs to be carried out to find out the exact effect of this amino acid change.

We were particularly interested in the few selected mutation hotspots only, as these regions tend to accumulate a high frequency of mutations compared to the other regions of the target genes. It is also true that other significant mutations can also be found in the other regions of the target genes. But in this study, we only wanted to check the profiles of the mutations which were previously reported to establish the already reported mutations as a biomarker for cervical cancer diagnosis. However, taking cue from our results, the full length of the target genes can also be taken into considerations for future studies. Furthermore, we had a limitation of smaller sample size and because of that no statistically significant association between the mutations and clinicopathological features of the patients was observed.

## Conclusion

Cervical cancer is a major cause of morbidity and mortality, particularly in developing countries. This study reveals that somatic mutations exist in cancer tissues of cervical cancer patients. Affected women are usually, working, and raising children, which creates substantial social problems. The data obtained from this study can be used to establish a mutation database for Bangladeshi cervical cancer incidents. Since the findings suggest that cervical cancer may harbor targetable oncogenic mutations, this should encourage further studies to better understand these mutations and exploit them for clinical use.

Nowadays, many cancer diagnoses apply specific mutation detection. Specific mutations for specific cancers are also being exploited for more tailored treatment strategies. Future studies are needed to validate this finding and to explore the biological and clinical importance of these mutations. Precise classification of cervical carcinomas in combination with mutation profiling is valuable for predicting disease outcome and may guide the development and selection of tumor-specific treatment approaches.

## Supplementary Information

**Additional file 1 Supplementary file 1:** Raw unprocessed gel images of the figures used in Fig. 1. PCR products were obtained using A. PIK3CA_1, B. PIK3CA_2, C. KRAS_1, D. KRAS_2, E. EGFR primer pairs. 50 bp ladder (Bioneer, USA) was used for comparison.

## Data Availability

The datasets used and/or analysed during the current study available from the corresponding author on reasonable request.

## Abbreviations

ATP: Adenosine triphosphate
BLAST: Basic Local Alignment Search Tool
*EGFR*: Epidermal Growth Factor Receptor
GTP: Guanosine triphosphate
HPV: Human Papillomavirus
*KRAS*: Kirsten Rat Sarcoma
NCBI: National Center for Biotechnology Information
NICHR: National Institute of Cancer Research and Hospital
*PIK3CA*: Phosphatidylinositol-4, 5-bisphosphate 3-kinase, catalytic subunit alpha
PtdIns: Phosphatidylinositols
