# Supplementary material for: Mutational profiles of marker genes of cervical carcinoma in Bangladeshi patients
Source: BMC Cancer. 2021 Mar 18;21:289. doi: 10.1186/s12885-021-07906-5 (PMC7977314; doi:10.1186/s12885-021-07906-5)
Supplement: Supplementary file 1 — Additional file 1 Supplementary file 1: Raw unprocessed gel images of the figures used in Fig. 1. PCR products were obtained using A. PIK3CA_1, B. PIK3CA_2, C. KRAS_1, D. KRAS_2, E. EGFR primer pairs. 50 bp ladder (Bioneer, USA) was used for comparison. [file 12885_2021_7906_MOESM1_ESM.pdf]

Supplementary file 1: Raw unprocessed gel images of the figures used in Figure 1. PCR products were obtained using **A.** PIK3CA_1, **B.** PIK3CA_2, **C.** KRAS_1, **D.** KRAS_2, **E.** EGFR primer pairs. 50bp ladder (Bioneer, USA) was used for comparison.

A.

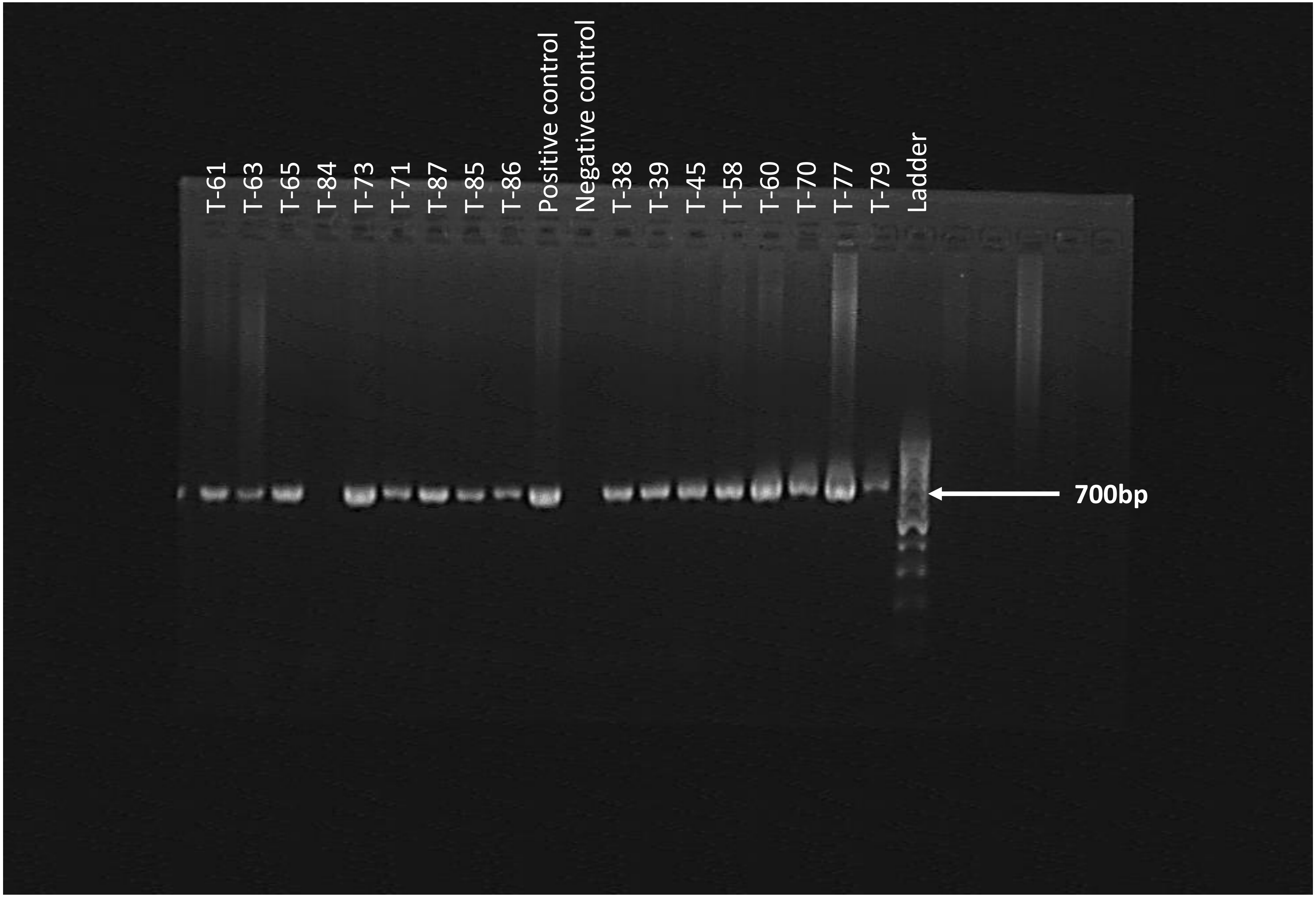

B.

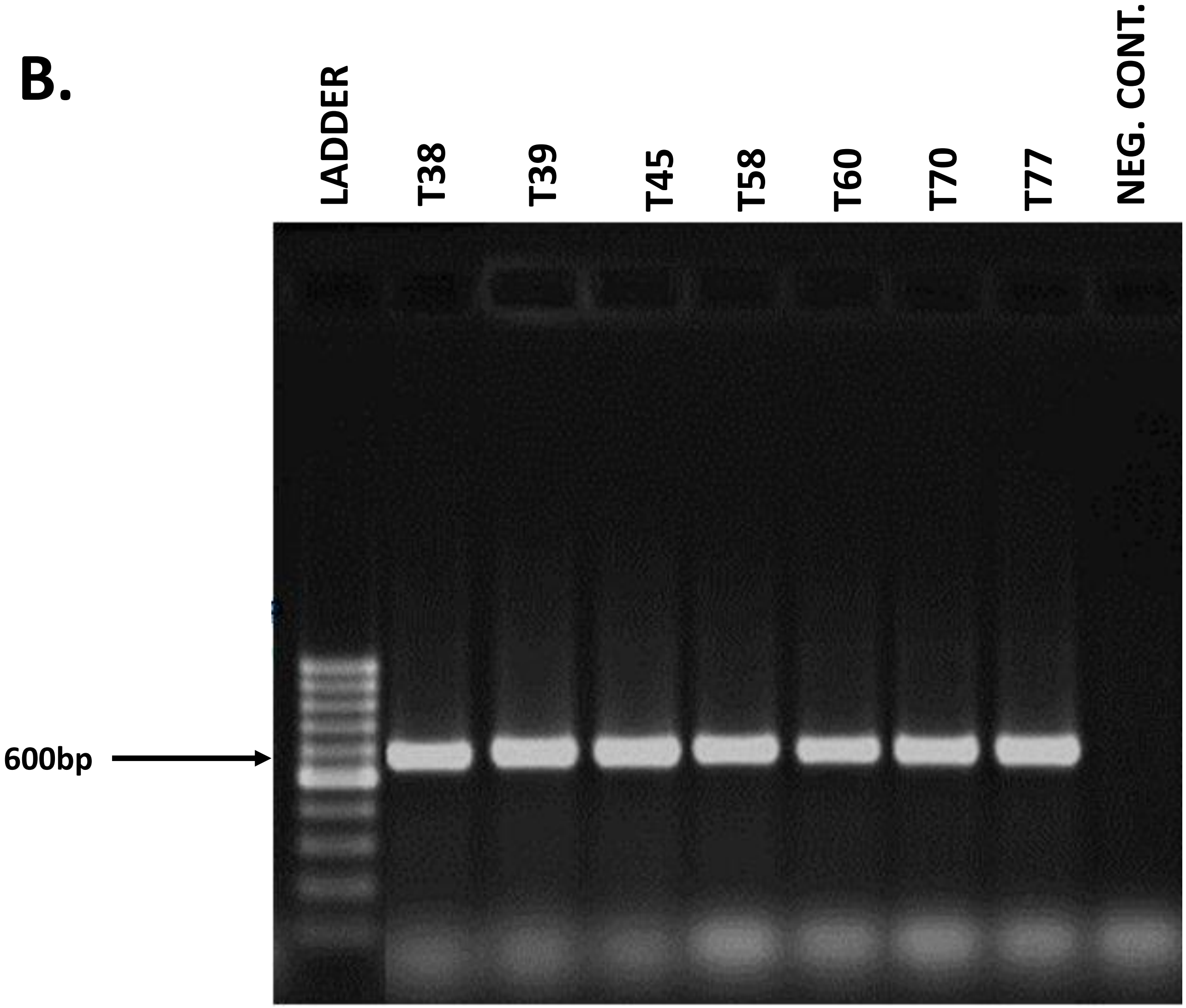

C.

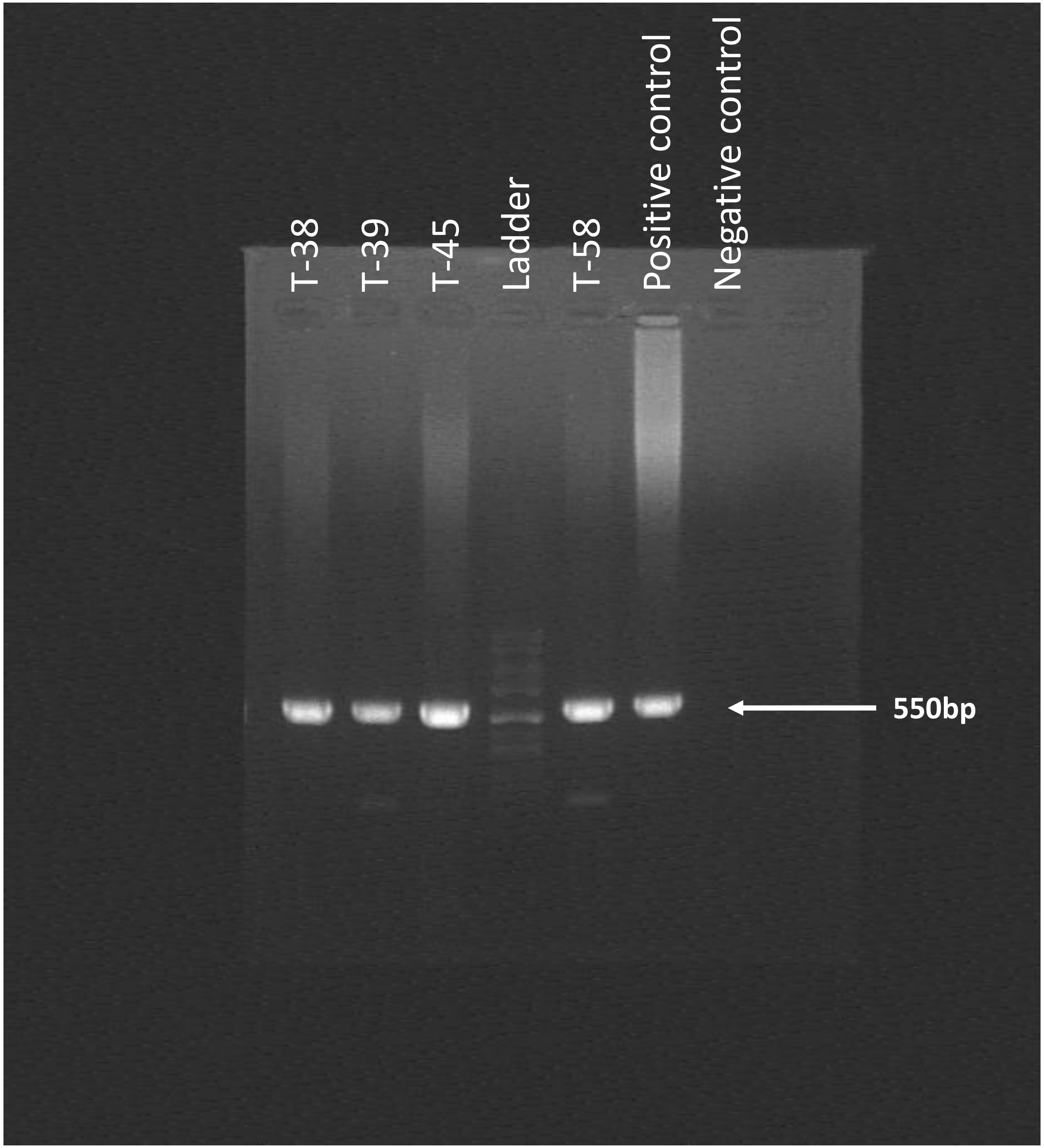
T-38
T-39
T-45
Ladder
T-58
Positive control
Negative control
550bp

D.

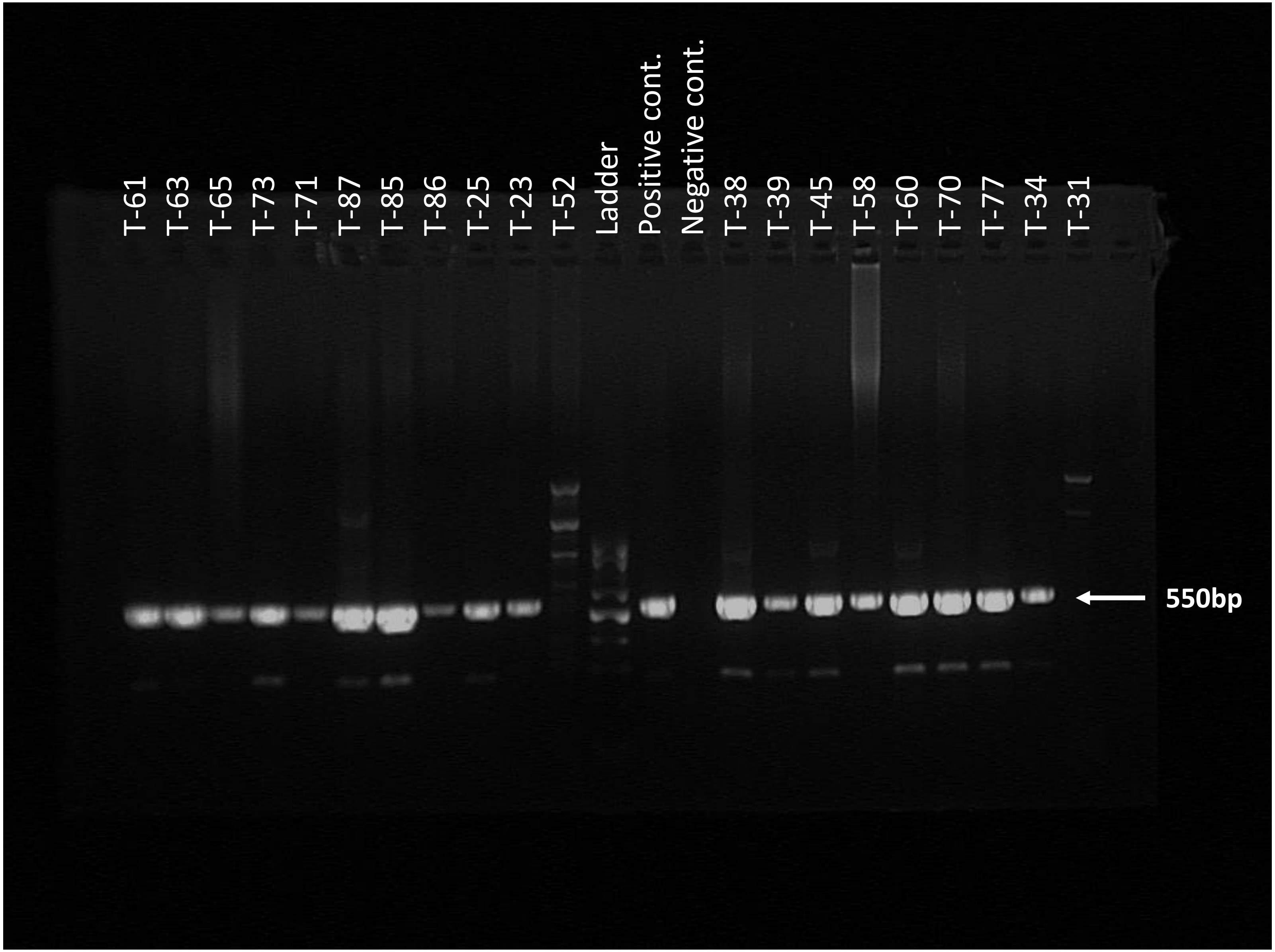
T-61
T-63
T-65
T-73
T-71
T-87
T-85
T-86
T-25
T-23
T-52
Ladder
Positive cont.
Negative cont.
T-38
T-39
T-45
T-58
T-60
T-70
T-77
T-34
T-31
550bp

E.

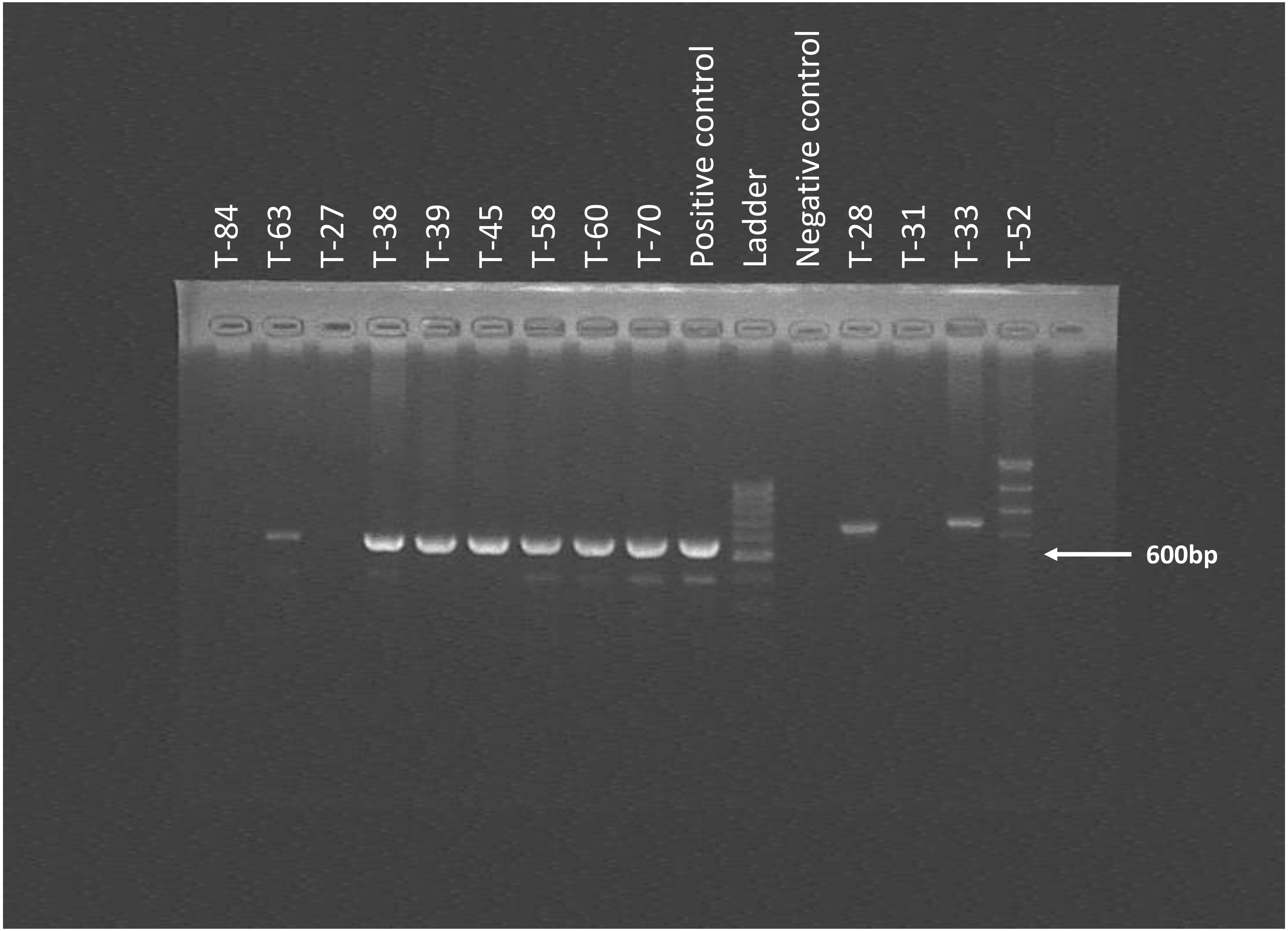

T-84
T-63
T-27
T-38
T-39
T-45
T-58
T-60
T-70
Positive control
Ladder
Negative control
T-28
T-31
T-33
T-52
600bp
